# MolBook UNIPI—Create, Manage, Analyze, and Share Your Chemical Data for Free

**DOI:** 10.1021/acs.jcim.3c00278

**Published:** 2023-06-26

**Authors:** Salvatore Galati, Miriana Di Stefano, Marco Macchia, Giulio Poli, Tiziano Tuccinardi

**Affiliations:** † Department of Pharmacy, University of Pisa, Via Bonanno 6, 56126 Pisa, Italy; ‡ Department of Life Sciences, University of Siena, 53100 Siena, Italy

## Abstract

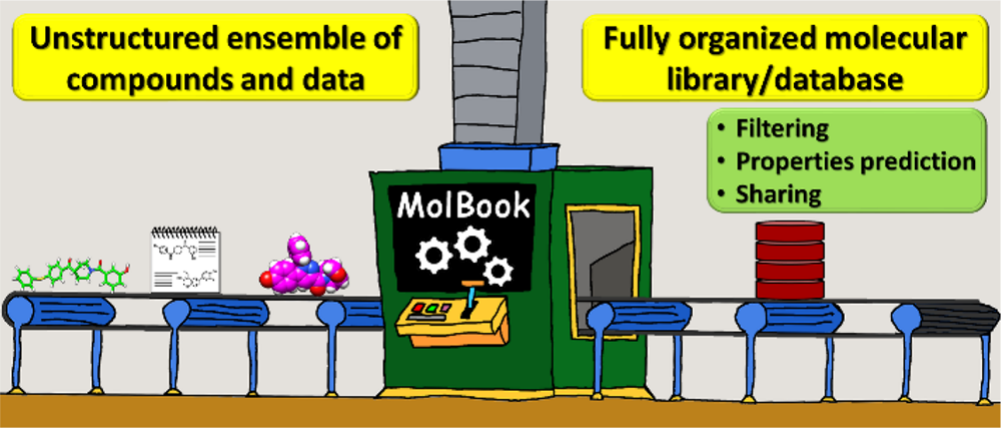

Here, we present MolBook UNIPI, freely available and user-friendly software specifically designed for medicinal chemists as a powerful tool for the easy management of virtual libraries of chemical compounds. With MolBook UNIPI, it is possible to create, store, handle, and share molecular databases in a very simple and intuitive way. The software allows users to rapidly generate libraries of bioactive ligands, building blocks, or commercial compounds by either manually creating single molecules or automatically importing compounds from public databases and pre-existing libraries. MolBook UNIPI databases can be enriched with all kinds of data and can be filtered based on molecular structures or properties, allowing the desired molecules, along with their structures and features, to be easily accessible in just a few clicks. Moreover, new molecular properties and potential toxicological effects of compounds can be rapidly and reliably predicted. Notably, all of these functions can be easily mastered even by inexperienced users, with no prior cheminformatics knowledge or programming skills, which makes MolBook UNIPI an invaluable tool for medicinal chemists. MolBook UNIPI can be downloaded free of charge from the project web page https://molbook.farm.unipi.it/.

## Introduction

In recent years, the management of large databases has become of primary importance, because of the massive amount of digitized data. The presence of databases in digital format allowed a rapid spread of techniques involving the search and analysis of stored data with the aim of obtaining useful information. Molecular databases, containing data for both synthetic and natural compounds with known activities for biological targets, have also been built for medicinal chemists and biologists, allowing experimental data to be shared quickly and in some ways automatically for the entire scientific community.^1^ The presence of these databases, most of which are freely available, facilitates the development of chemoinformatics. This discipline involves the management of chemical data and its processing in order to perform operations of a complex nature; for instance, typical chemoinformatics approaches involve similarity searching, where the structures of millions of molecules are compared in order to identify those that have the desired similarity with respect to the reference molecule used.^2^ Recently, the widespread use of artificial intelligence (AI) methods, together with the use of big data, allowed the development of predictive models that provide insights into various properties of small molecules.^3^ In this area, the prediction of the toxicological profile of chemical compounds has become crucial for assessing their safety with the aim of reducing animal testing, which has a great economic and ethical impact on research.^4,5^ The increasing digitization of data has also led to the development of tools to help manage this information. Important examples include the RDKit library of Python, C, and Java wrappers for deep and complex chemoinformatics operations. The functions contained in RDKit^6^ can be integrated into workflows for fast and reusable data processing, thanks to their implementation into KNIME software (in the form of KNIME nodes).^7^ Furthermore, the developed MolFrames library provides the possibility to manage molecular databases working with pandas dataframes. Precisely, MolFrames allows the user to search and visualize the molecular structures through code written and integrated in JupyterNotebook. An example for the integration of RDKit functionality is the MayaChemTools package, a collection of Perl and Python scripts, modules, and classes to support computer-aided drug design.^8^ In a similar way to the RDKit library, OpenChemLib is a Java-based framework that provides core cheminformatics functionality and user interface components. Its main focus is on organic chemistry and small molecules. Bioclipse is another advanced open source framework that includes chemoinformatics and bioinformatics tools.^9^ Bioclipse represents a valuable tool for processing chemical data, allowing the user to employ scripting languages to quickly execute custom commands. In this context, it is worth mentioning DataWarrior as one of the most complete and multifaceted pieces of open source software for chemical data management and visualization.^10^ The software provides the user with several features that can be applied for chemical data analysis and processing. The embedded *in silico* tools allow the prediction of chemical and biological properties of molecules useful for filtering chemical structures and to perform several types of chemoinformatics and structure-based analyses. Such features make DataWarrior one of the most powerful pieces of software for chemical data management. However, although they allow multiple and useful analyses and evaluations, we believe that these tools are more oriented to computational chemists, or at least to users with a certain level of experience in programming and/or chemoinformatics. For instance, RDKit certainly requires programming skills for its application, since it is a code wrapper. Moreover, while Bioclipse and DataWarrior have valid graphic user interfaces, they are not always straightforward to use, and many of the available features, such as chemical space evaluation and visualization through multiple descriptors, principal component analysis, and self-organizing maps, as well as multivariate/multidimensional structure–activity or structure–property evaluations, may not actually be useful for medicinal chemists or biologists looking for simple chemical database management software. In light of these considerations, we believe that the availability of simple, intuitive, and user-friendly chemical data management software is essential for users who do not have programming skills and have never or rarely approached chemoinformatics but still need to create and manage databases of chemical compounds. In this context, we have developed MolBook UNIPI, user-friendly and freely accessible software that allows the management and analysis of chemical data, including the possibility of predicting molecular properties. The design of MolBook UNIPI is intended to provide the user with an intuitive tool by means of which operations can be performed in a fast and very simple way. Users can create their own databases (herein referred to as projects) in which they can add and import data and structures of chemical compounds. Projects can be saved and easily shared, as they are stored as simple folders. The data contained in the projects can be exported to formats commonly used by applications such as Microsoft Excel; furthermore, project directories can also be shared between different users, thus allowing multiple access to the system. MolBook UNIPI includes several features that facilitate property-based and/or structure-based searches, thereby enabling the efficient handling of extensive databases and easy retrivial of necessary information. The implementation of the VenomPred platform,^11^ recently developed by our team, provides the user with a valuable tool for predicting the toxicological profile of stored molecules. The aim of MolBook UNIPI is to be simple and intuitive, while providing the possibility to perform complex search and analysis tasks. The user has the possibility to take advantage of the various tutorials available on the official MolBook UNIPI Web site (https://molbook.farm.unipi.it/howto/), which contains a form for reporting bugs and feedback that will be fundamental for future development of the software, in order to improve its usefulness for the scientific community.

## Results

The key points that inspired the creation of MolBook UNIPI are (a) the lack of free software for chemical data management and (b) the importance of making the procedures for database management user-friendly by providing the nonexpert user with a simple and intuitive tool. The main window of MolBook UNIPI is shown in Figure S1. The user can find all functions in the software menus, but the most useful ones are also accessible as icon buttons in the software toolbar. The basic concept of the software consists of creating projects to store molecular data and carry out operations such as data import/export and/or compounds filtering. In this section, we aim to demonstrate the potential usefulness of MolBook UNIPI by describing three application cases of the software: case A, creating and exploring a project from scratch; case B, query, analysis, and prediction of properties; and case C, filtering of natural compounds (within the Supporting Information).

### Case A: Creating and Exploring a Project from Scratch

**Figure 1:**
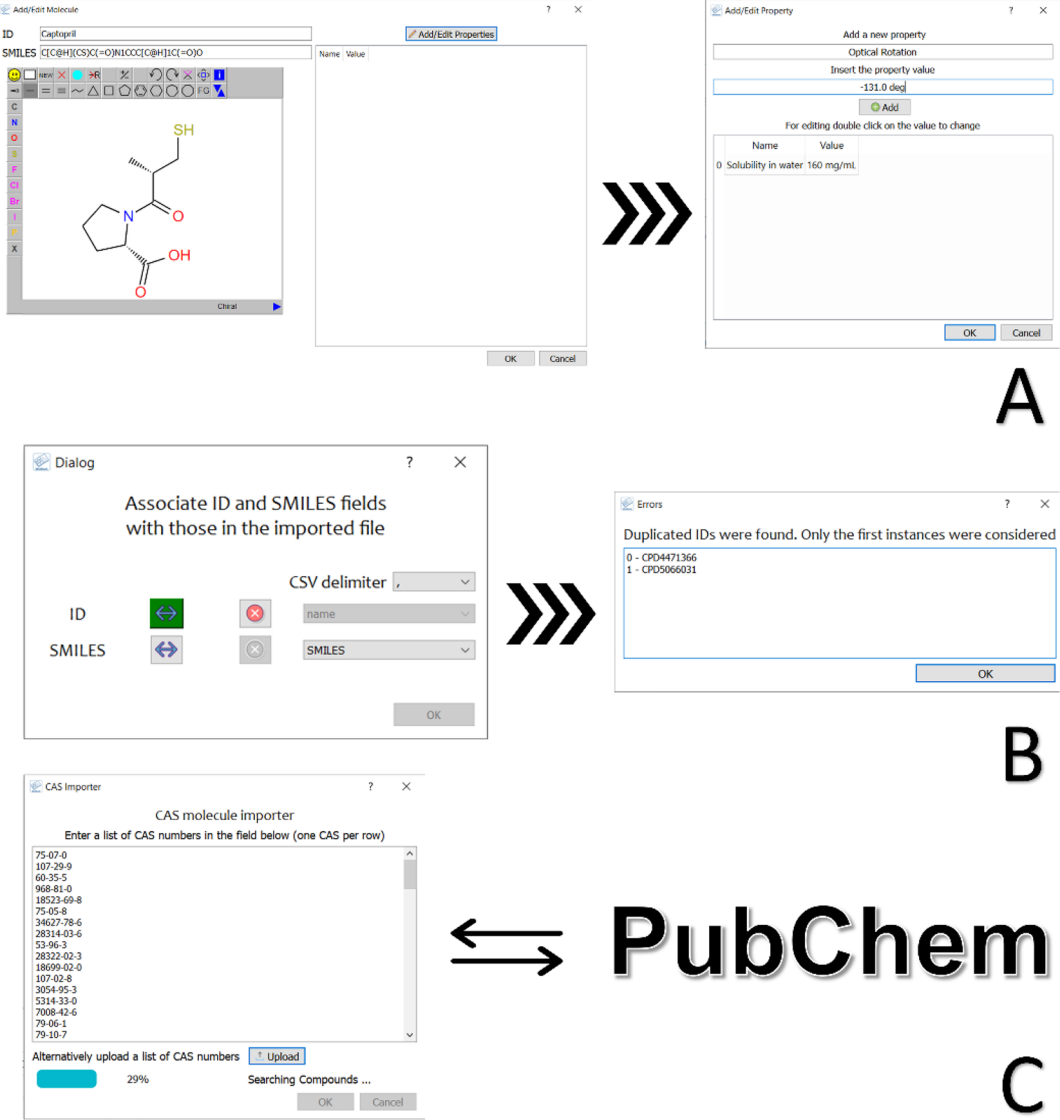
Different methods for adding data to the project: by manual sketching (A), by importing external files (B), and by retrieving the chemical structures from the CAS number via the PubChem database (C).

MolBook UNIPI projects can be created using the “New Project” function from the file menu or with the corresponding folder icon in the toolbar (Figure S1). Projects are displayed in individual tabs accessible from the main window. The software supports the management of multiple simultaneously opened projects; however, the executed tasks affect only the current active tab. A project tab contains a table whose purpose is to display and select the stored data. When creating a new project, the table appears empty, and the user must add data to update it. Data can be added in multiple ways; here, we explore all solutions available to the user. If the user intends to add data manually, then it is necessary to draw the chemical structure of the desired compounds and add the properties of interest. The addition of a compound can be done using the *Add Molecule* function within the Edit menu, which opens the window containing the JSME sketcher and the property table (Figure 1A). JSME sketcher allows users to draw chemical structures in a user-friendly way, providing the user with several preset chemical fragments.^12^ The sketcher is linked to the SMILES (Simplified Molecular Input Line Entry System) field so that the SMILES notation of the drawn structure is simultaneously shown in the corresponding text box. Conversely, the SMILES box can be used to directly enter the SMILES of the compound to be added; in this case, the JSME sketcher will automatically display the structure of the compound. The panel adjacent to the sketcher allows the user to include properties associated with the molecule entry. Adding/editing properties is performed by clicking the “Add/Edit Property” button, which opens the corresponding window where the user must specify the name and value of the property. If properties are already present (as in the case of editing an existing molecule entry), then their value can be changed by double-clicking on the displayed table. The property table proves useful for storing relevant data associated with the compounds, such as biological activity (for bioactive molecules), information on the available quantity, source vendor (for commercial compounds), as well as any relevant comment.

In case the data to be uploaded are already stored in a digital format, such as Microsoft Excel (*.xlsx), Comma-Separated Values (*.csv), or Structure Data File (*.sdf), the software provides the option of importing these data directly into the project. Data can be imported either into an empty project or into a one that already includes entries. The import function is accessible from the “Import” option located in the Database menu (Figure S1). The “Import” window (Figure 1B) requires the selection of fields that include molecule ID and structures in SMILES format. For csv files, the separator can be specified from the corresponding drop-down menu. MolBook UNIPI does not handle duplicate instances in terms of compound ID; therefore, if duplicates are encountered, only the first instance is considered. Nevertheless, duplicate IDs are noted in an additional message window. Finally, the software allows the user to import chemical structures directly from the CAS number. This function employs the PubChem database^13^ to first match the CAS identifiers with SMILES notations and then download the data by incorporating them into a MolBook UNIPI project. The “*Import from CAS number*” option is located in the Database menu of the main window (Figure S1). The “CAS importer” window has a blank box for entering CAS numbers (Figure 1C); additionally, if the user has a list of CAS numbers as a txt file, this can be loaded directly with the corresponding button. Searching for structures with the CAS identifier requires an Internet connection in order to query the PubChem database. Each compound downloaded includes the IUPAC name, which is displayed as a property associated with the molecule entry. This function proves very useful when building a database of reagents, building blocks, or known compounds to be used for chemical synthesis.

Finally, it is worth mentioning the possibility of including external files as attachments to each molecule entry with the Chemical Notebook function. The Chemical Notebook is accessible by means of the context menu that can be opened by right-clicking on a molecule entry within the table. The corresponding window allows users to add, open, and delete attachments for the molecule under consideration. Such attachments can be, but are not limited to, images, PDF, and text files. The presence of one or more attachments for a given compound is indicated by the presence of the paperclip in the column named with “#”.

When creating a project within MolBook UNIPI, the user can visualize the data through two different modes: Classic and Table Views (Figure S2).

Classic mode (Figure S2A) presents a scrollable project table containing the data, with only the structure of the selected molecule entry displayed at the top of the table. Table View (Figure S2B) mode allows the structure of the compounds to be displayed directly in the corresponding rows of the table. Specifically, the field containing the SMILES string of each molecule in Classic View mode is converted to an image showing the 2D chemical structure. Classic mode is suggested in the case where the user is interested in browsing item data by giving attention to the individual properties of the stored molecules, while Table View mode is recommended for a visualization focused on the structure of the compounds. Both modes are dynamically interchangeable and allow for individual rows to be highlighted. This function can be performed by opening the context menu and selecting the desired color (green, yellow, or red). The selected rows will assume the chosen coloring, as shown in Figure S2A, which can be removed through the same menu at any time. This function can be useful for quickly identifying molecules of particular interest to the users.

### Case B: Query, Analysis, and Prediction of Properties

MolBook UNIPI is designed with the goal of providing the user with an efficient and powerful tool for managing chemical data, and for this reason, it includes several features for analyzing the chemical compounds stored within projects. In this test case, the various features discussed will be applied to a MolBook UNIPI project containing a random set of 2000 compounds downloaded from the ZINC database.^14^ The analysis of chemical compounds is commonly based on the physicochemical properties associated with the structure of molecules. MolBook UNIPI allows the user to compute molecular weight, number of heavy atoms, chemical formula, LogP, and several other chemical properties of compounds within the project using the built-in RDKit features.^6^ These functions are easily accessible directly from the Database menu of the software (Figure S1). During the calculation phase, all compounds are processed, and the selected properties are calculated and then stored as numerical values/strings within new columns that are added to the table. This process updates each molecule entry by adding the calculated properties to the corresponding property columns, which can also be edited for any future corrections by the user.

Since each data item in the table columns can be sorted dynamically, the user has the option of focusing on compounds with a desired range of property values, such as molecular weight or LogP. Given the importance of evaluating the toxicity profile of chemicals, the VenomPred tool was included in the software. VenomPred is a platform developed by our team to assess the potential mutagenic, carcinogenic, estrogenic, and hepatotoxic effect of chemical compounds. Such a platform is available online as a freely accessible web tool. On the other hand, with MolBook UNIPI, users do not need Internet access to obtain VenomPred predictions, which can be performed directly in the software with the appropriate window. Similarly to the previously calculated properties, the toxicity profiles are reported as numerical values in a range between 0 and 100, which are saved in new columns as additional properties associated with the compounds (Figure S3).

The ability to calculate and predict the properties of compounds is a powerful tool for obtaining a comprehensive overview of the data stored within the MolBook UNIPI project. Among the features of the software, the possibility of querying the database (represented by the project) can be applied to retrieve only those compounds that exhibit certain properties. In line with what is indicated in the software, project data can be filtered based on the properties associated with individual molecule entries. With this feature, compounds can be filtered based on the values contained in the IDs/SMILES column or in the columns that correspond to the properties added and/or predicted by the user. The “*Query Properties*” window allows the selection of the column (property) to be considered for filtering, the type of comparison, and the reference value. In terms of comparison, eight options are available for applying filters. Specifically, a property can be queried by comparing its value numerically (≥, >, <, ≤), by exact match (equal or different) and, last, whether or not the string value is contained in the property. Figure S4 shows an example of a property query in which the user searches for compounds with a molecular weight greater than or equal to 300, LogP lower than or equal to 2.0, and containing the nitrogen symbol in the chemical formula. The application of such a query in the example project provides a new project containing only those compounds that meet the filter criteria, as represented in Figure S4.

A second method of querying the project is by “Structural Query”, which filters compounds based on their structure, therefore from a chemical point of view. This feature is particularly useful for searching for compounds with the desired degree of similarity with respect to a reference molecule or for identifying chemicals that have a specific substructure. Figure S5 shows an example of a “structural query” in which the user retrieves compounds presenting a coumarin core. The filter results can also be easily analyzed with the GridView function available in the software to directly display the chemical structures in a grid table.

A third application of this filter is the similarity search, which allows the identification of compounds similar to a reference molecule, which can be a valuable tool for performing a SAR analysis on bioactive compounds. The structural queries require more machine time than property queries due to the conversion of compounds into RDKit objects to carry out similarity comparisons or substructure/superstructure matchings. The timing is, of course, strictly dependent on both the amount of data within the projects and the hardware settings of the executing machine; however, for databases with less than 100 000 molecules, a structural query requires only a few seconds.

## Discussion

MolBook UNIPI allows users to create, manage, and analyze chemical databases. The software is developed to provide a user-friendly tool even for nonexpert users, given the lack of free programs of this type. It supports several methods for creating a database: manual addition by drawing compound structures, importing external files (e.g., xlsx, csv, and sdf files), and retrieving data from PubChem. Databases can be managed directly within MolBook UNIPI; moreover, they can be exported to formats accessible with external software (such as Microsoft Excel) for easy sharing. The program includes functions for querying the database in order to identify compounds with certain properties or specific chemical structures. With the latter functionality, the user can perform searches even on large databases with the aim of identifying compounds with a certain degree of similarity to the reference molecule or retrieving molecules that have a certain substructure. The ability to predict the chemical properties and toxicological profile of molecules makes MolBook UNIPI a valuable resource for medicinal chemists and biologists. Furthermore, MolBook UNIPI is designed to allow project sharing among collaborators. By using a file sharing software tool, such as Google Drive or OneDrive, and sharing the project directory, different users can work on the same project; nevertheless, in order to prevent sharing issues, in case several users open the project at the same time, only the first user will maintain the Read/Write mode access, whereas all other users will receive a notification indicating which user is active in Read/Write mode and suggesting to use the Read only mode.

### Future Perspectives

Our team is focused on keeping MolBook UNIPI updated by including new features and improving existing ones. Therefore, feedback from the scientific community is essential to update the features and include new functionalities that are useful to the users. In this context, we are currently updating the VenomPred platform for *in silico* toxicological predictions to allow the possibility of assessing new types of toxicity end points, as well as achieving an improved predictive performance thanks to an optimized exhaustive consensus prediction strategy. These new features will be included in new versions of MolBook UNIPI. Similarly, the development of machine learning models for the prediction of chemical and biological properties will be integrated into the software to assess aqueous solubility and biological membrane permeations. To facilitate the users in the field of synthetic chemistry, a tool for searching for compounds within databases of commercial suppliers will be introduced in new versions of the software. Such a tool may be useful for purchasing reagents and chemicals, avoiding multiple searches on different Web sites, and providing an overview of available options. It is worth mentioning that a tool for providing information on the synthesis of a molecule will be developed and incorporated into MolBook UNIPI. This function will be profitable for synthetic chemists, given the importance of gathering information on reactants and synthesis conditions. A similarity search function will be included to retrieve compounds from the public ChEMBL database that are structurally similar to a reference query molecule in order to obtain an overview of structurally related molecules already present in the literature. Likewise, a similarity search within the RCSB database^15^ will be made available, with the aim of identifying ligands similar to the query molecule whose X-ray structure in complex with a biomolecule is deposited therein. Finally, the project export options will be extended with the possibility of saving databases as MS Word tables, while a 3D viewer for visualizing a reliable, low-energy conformation of the database molecules will be implemented in the next versions of MolBook UNIPI.

## Conclusions

We have developed new user-friendly standalone software for creating, managing, and analyzing chemical databases. This software is freely available to the scientific community and therefore represents a useful tool for easily handling chemical data. MolBook UNIPI allows quick and easy query operations to search for compounds of interest based on properties and structural features. Properties of molecules within the databases can be calculated, whereas toxicological profiles can be predicted with the built-in VenomPred platform. Users can freely download the software from the project webpage https://molbook.farm.unipi.it/, where comprehensive documentation including tutorials is provided. On first startup, the software runs with a demo mode of 15 days; at the end of the trial period an activation code will be requested to continue using MolBook UNIPI. This activation code is provided free of charge and can be requested using the appropriate form on the official Web site. The Web site includes a support form through which users can submit feedback regarding software bugs and suggestions that will be handled by our team.

## Data Availability

MolBook UNIPI is freely distributed at the following link: https://molbook.farm.unipi.it/download/. It can be downloaded for Linux, MacOS or Windows. Tutorials on the use of MolBook UNIPI, which include instructions for creating a project, importing data, and querying a database, are available at the following link: https://molbook.farm.unipi.it/howto/. The graphical user interface of MolBook UNIPI is built with Qt through the freely accessible Qt Designer software. The source code is written in the Python language. The current version of MolBook UNIPI runs under Python version 3.8.13. The data are stored in pandas dataframes, a freely available Python library. RDKit functions are used for handling and representing chemical structures. JSME, a free molecule editor, is incorporated as a sketcher of molecular structures. Access to the PubChem database (https://pubchem.ncbi.nlm.nih.gov/) is provided by the open-source library PubChemPy. For toxicity predictions, the machine learning models of the VenomPred platform (http://www.mmvsl.it/wp/venompred/) are included in the software through the free scikit-learn library. Executable versions of MolBook UNIPI are generated with the freely accessible PyInstaller library. Installers for Windows systems are created with the free Inno Setup software. Installers for MacOS systems are created with the free WhiteBox Package software.

## Supporting Information Available

(Figure S1) Main interface of MolBook UNIPI; (Figure S2) MolBook UNIPI visualization modes; (Figure S3) calculation and prediction of properties; (Figure S4) property query functionality; (Figure S5) structural query functionality; case C: filtering of natural compounds; materials and methods; references (PDF)
