# Supplementary material for: MolBook UNIPI—Create, Manage, Analyze, and Share Your Chemical Data for Free
Source: J Chem Inf Model. 2023 Jun 26;63(13):3977–82. doi: 10.1021/acs.jcim.3c00278 (PMC10336919; doi:10.1021/acs.jcim.3c00278)
Supplement: Supplementary file 1 — ci3c00278_si_001.pdf [file ci3c00278_si_001.pdf]

Supporting Information

MolBook UNIPI - create, manage, analyze and share your chemical data for free

Salvatore Galati[1,*], *Miriana Di Stefano*[1,2], *Marco Macchia,*[1] *Giulio Poli*[1] *and Tiziano* Tuccinardi[1,*]

[1] Department of Pharmacy, University of Pisa, Via Bonanno 6, 56126 Pisa, Italy. [2] Department of Life Sciences, University of Siena, 53100 Siena, Italy

Address correspondence to: Salvatore Galati, salvatore.galati@phd.unipi.it; Tiziano Tuccinardi, tiziano.tuccinardi@unipi.it

Table of Contents

Figure S1. Main interface of MolBook UNIPI S2

Figure S2. MolBook UNIPI visualization modes S2

Figure S3. Calculation and prediction of properties S3

Figure S4. Property query functionality S3

Figure S5. Structural query functionality S4

Case C: Filtering of natural compounds S5

Materials and Methods S7

References . S13

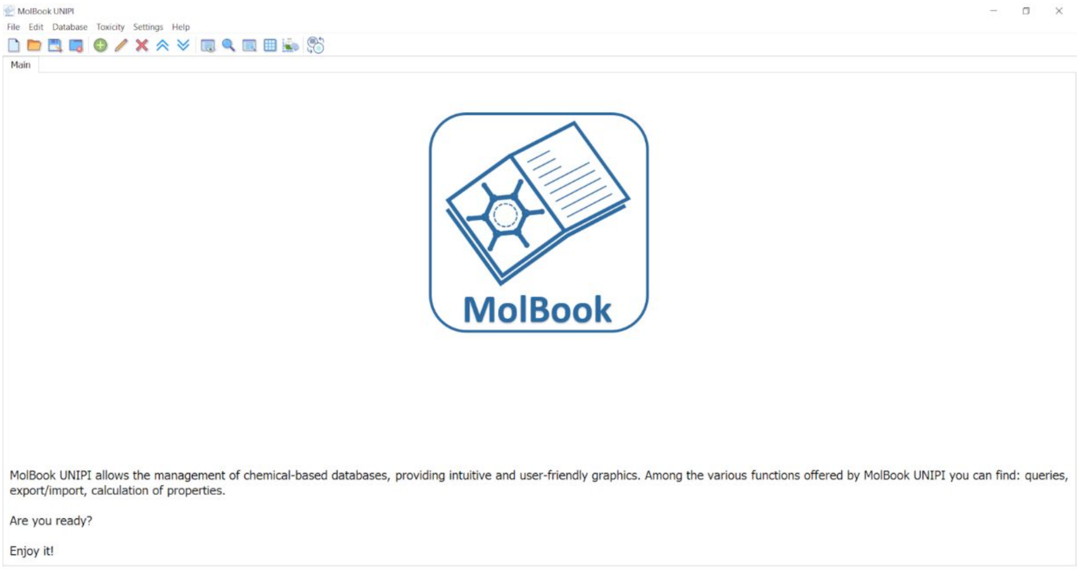

Figure S1. Main interface of MolBook UNIPI.

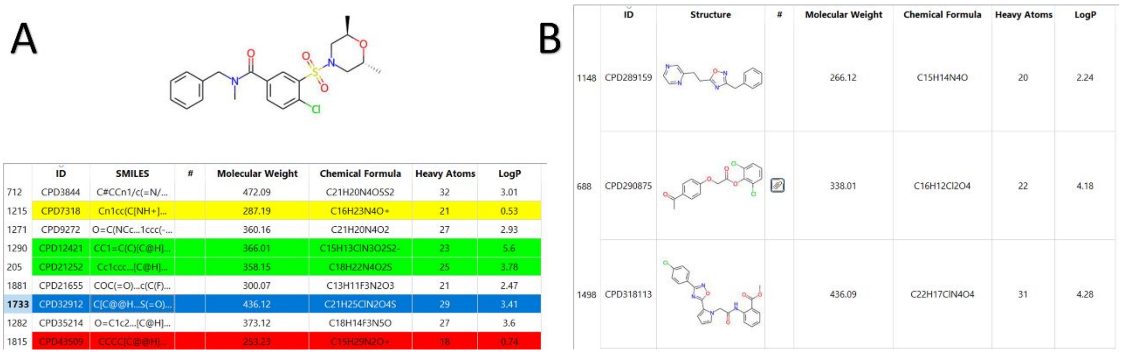

Figure S2. MolBook UNIPI visualization modes. (A) Classic mode, showing only the structure of the selected table row, and (B) Table View, in which the structures of compounds are shown within the corresponding table rows.

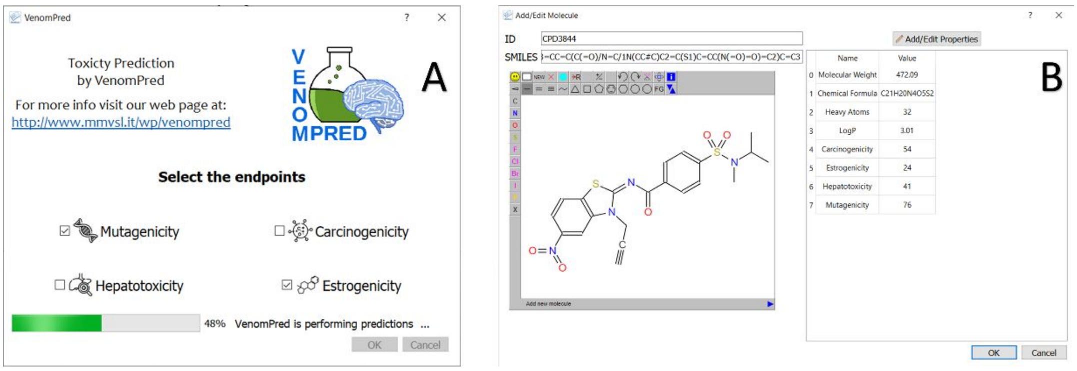

Figure S3. Calculation and prediction of properties. Figure S3A represents the VenomPred tool for toxicity predictions, while Figure S3B shows a molecule entry with several computed properties.

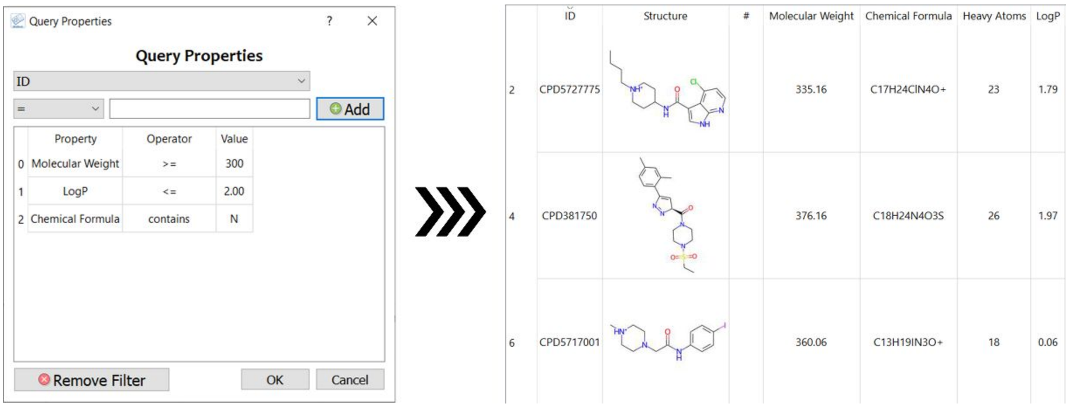

Figure S4. Property query functionality. Application of an example query for searching for compounds on the basis of molecular weight, LogP and chemical formula.

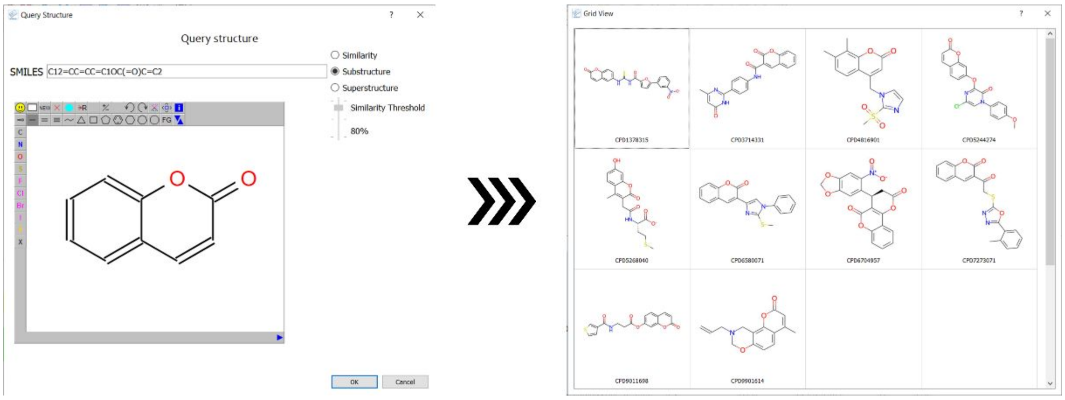

Figure S5. Structural query functionality. Application of an example query for searching for compounds presenting a coumarin core by employing the substructure option.

Case C: Filtering of natural compounds

From a medicinal chemistry point of view, the evaluation of the structure of the molecules in the optic of future pharmaceutical development is essential. This evaluation includes analyses to identify and avoid compounds that may generate interference during their biological assessments, while prioritizing those showing a promising potential to become candidate drugs. In this context, we propose the analysis of a set of 1000 compounds obtained from the public COCONUT[1] database in order to obtain molecules with a suitable drug-like structure. The uploaded database consists of naturally derived chemicals with different structural motifs and properties. MolBook UNIPI allows the evaluation of the drug-likeness of chemical structures according to Lipinsky's rule of 5 (RO5). Specifically, four chemical properties (hydrogen bond acceptors and donors, molecular weight and LogP) are calculated for a given molecule, and it is checked whether compounds satisfy the corresponding criteria. The function for performing the RO5 analysis can be found in the Database menu of the software. Through this function, each molecule in the project is evaluated and a new column containing the results of the analysis is created in the main table. For molecules that do not match all criteria, the ”Not Passed” flag is shown along with the reasons for the mismatch. In order to obtain only compounds that meet all RO5 criteria, the project can be subjected to a property-based query as reported in Case study B (Figure S4). In particular, the query will be applied to the property called "Drug-likeness (RO5)" to retrieve compounds with the "Passed" flag. MolBook UNIPI also includes the functionality to evaluate if a molecule could generate interference when subjected to biological assays. This analysis is based on a search for substructures reported as potential cause of pan-assay interference. Compounds presenting such substructures, which could thus behave as pan-assay interference compounds (PAINS),[2] can be identified using the built-in “Calculate PAINS” tool present in the database menu under PAINS filer. The corresponding window allows user to select the evaluation of the molecules according to three different filters that can be run simultaneously. The result of each filter will be stored in the newly generated columns of the main table. Each result will contain the number of interfering substructures detected in the

molecules. In this context, examining the molecular moieties that generated a PAINS alert is essential in order to understand the results. For this purpose, the PAINS mapping tool provides a grid view interface where the user can select the PAINS filter to highlight the matching substructures that have generated alerts in the analyzed molecules. The tool allows users to switch between the PAINS A, B and C filters, highlighting the different groups of PAINS substructures with different colors (see Figure S6). Furthermore, the selected structures of the grid view interface are automatically selected into the project table facilitating the user to export or delete only the desired entries.

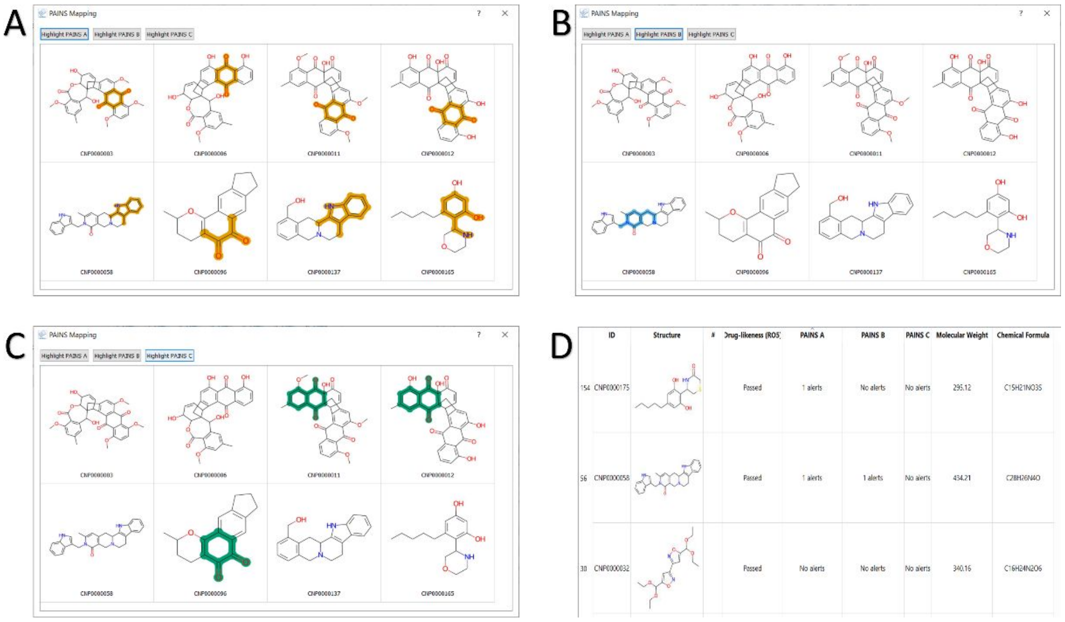

Figure S6. MolBook UNIPI functionality for PAINS mapping and drug-likeness filtering. The PAINS mapping tool highlights the substructures matched by the filters: PAINS A (A), PAINS B (B), PAINS C (C). The results of both PAINS and drug-likeness analysis are consultable thorough the table view (D).

MATERIALS AND METHODS

MolBook UNIPI is a standalone desktop software developed in the Python programming language version 3.8.13 that uses the Qt platform for creating graphical user interfaces (GUIs). The first public version of MolBook is available for Windows and Linux operating systems. The software's GUIs are designed with the Qt Designer software and integrated into python code via the pyqt5 library. MolBook UNIPI is based on the RDKit chemoinformatics and the pandas database management library. RDKit provides several functions for the design and management of chemical structures. MolBook UNIPI uses the RDKit library to represent the structures of compounds and for conversions into the specified file formats during data import/export. Internally, MolBook UNIPI uses a pandas dataframe as a container to hold molecule data. A pandas dataframe is easy to handle and allows direct implementation with the pyqt5 library functions. The software is equipped with an error-handling system that prevents it from crashing due to bugs present in the source code and allows it to keep running properly even after an error event. Secondly, the user can report to our team the error shown within the pop-up window (Figure S7), which is displayed in case of errors, using the form available on the official MolBook UNIPI website.

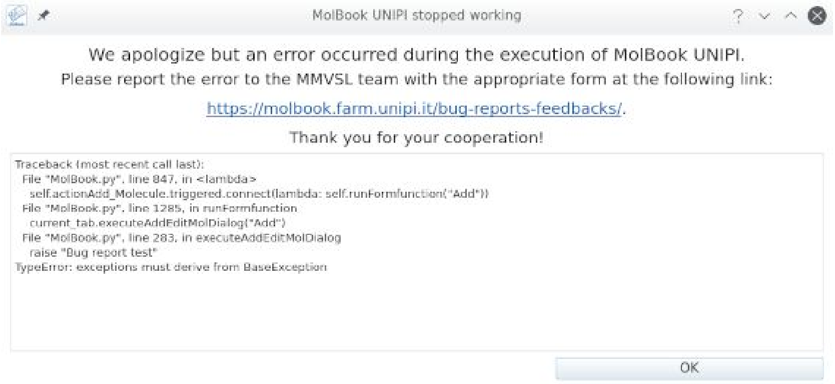

Figure S7. MolBook UNIPI error handler window.

Chemical structure processing

MolBook UNIPI relies on the RDKit library for loading and handling molecular structures. In particular, the software accepts input structures as *sdf* or *csv/xlsx* files, the latter containing a column with SMILES notations (see *Data management* for more details). These data are loaded with RDKit's dedicated function that internally generates the molecular graph containing all the information on the atoms of the compounds. In all cases, the compounds are converted into RDKit mol objects and subjected to a "sanitization" protocol.[3] This procedure is often used to ensure the consistency of the chemical structure in order to avoid atoms with undesirable hybridizations or unusual bonds. The result of the sanitization is a molecular graph that can be represented with Lewis dot structures complete with octets. The steps involved in the protocol scheme and their description are reported in Table S1.

Table S1. Performed steps of the RDKit sanitization protocol.

Operation	Description
clearComputedProps	removes all calculated properties of the molecule and its atoms and bonds.
cleanUp	standardizes non-standard valence states
updatePropertyCache	calculates the explicit and implicit valences on all atoms
symmetrizeSSSR	calls the symmetrized smallest set of smallest rings algorithm
Kekulize	converts aromatic rings to their Kekule form
assignRadicals	sets the number of radical electrons (if any) on each atom
setAromaticity	recognizes aromatic rings and ring systems (see above), sets aromatic flag on atoms and bonds
setConjugation	determines which bonds are conjugated
setHybridization	computes the hybridization state of each atom
cleanupChirality	removes chiral tags from atoms that are not sp3 hybridized

adjustHs	includes explicit hydrogens where necessary to preserve the chemistry integrity.

<u>*Data management*</u>

The working system of MolBook UNIPI is based on the creation of projects that are graphically displayed as tabs within which a database of molecules is hosted. The users can switch among different projects while keeping data separate and performing several tasks, which are described below. Each individual project can be saved to a path specified by the user. Specifically, a MolBook UNIPI project is stored as a folder including all information needed for consultation. Saved projects can be either accessed with the incorporated *project load* function, which requires manual selection of the project folder by the user, or directly loaded using the *recent projects* submenu. The compound data are displayed in a table that allows user to quickly consult the chemical structure and associated information. In particular, each row in the table is associated with a molecule with an identifier ID that must be unique, while the structure of the molecule is stored as 2D representation encoded by SMILES notation. Two options are available for displaying the data: the "*Classic*" view, which exhibits one molecule structure at a time, and the "*Image Table*" mode that displays each structure directly in the corresponding table row, thus facilitating the visualization of multiple compounds. The above described view modes are dynamically interchangeable, giving the user the possibility to choose the preferred one according to the circumstances. Three different resolution sizes are available and can be selected by the user. In addition, MolBook UNIPI includes a "*GridView*" mode that allows users to visualize the structures of selected molecules by opening an external window. The structures of the molecules are displayed inside cells of a grid with their unique identifiers.

A dataset can be created from scratch by adding compounds individually. Indeed, the JSME molecular sketcher was included in the application to draw chemical structures. JSME is a free molecular editor written in JavaScript that supports the drawing and editing of molecules. Adding a

molecule in a MolBook UNIPI database includes the possibility of saving additional information by linking it to the molecule entry. Similarly, the editing of a stored compound is easy: users can change the chemical structure by directly employing the JSME sketcher or update the desired properties. MolBook UNIPI is endowed with functions that provide the users with the possibility of importing/exporting their own data. In particular, a MolBook UNIPI project can be created by importing data already saved in Microsoft Excel (*.xlsx), Comma-Separated Values (*.csv) and Structure-Data File (*.sdf) formats. For the *xlsx* and *csv* formats, the user is required to select the columns containing the unique IDs of the compounds and their relative structures stored as SMILES strings. For the *sdf* format, only the field representing the ID of the compounds has to be selected. Concerning the export, the same format types are available. MolBook UNIPI stores chemical structures as images instead of SMILES strings when exporting data as *xlsx* files; this option allows users to create Microsoft Excel tables with a visualization similar to that provided by MolBook UNIPI with the "*Table View*" mode. A further feature for importing data into a MolBook UNIPI project is provided by a tool that allows loading molecules by indicating their CAS number. The tool uses the functions of the PubChemPy library, giving the possibility of searching the PubChem database for retrieving the desired compounds; thus, for the matched CAS numbers, the corresponding chemical structures and IUPAC names are downloaded and incorporated directly into the project. MolBook UNIPI projects already saved by the user include the possibility of removing molecule entries and properties, making the user's database fully editable. Individual entries can be associated to external files with the embedded "Chemical Notebook" function. The "Chemical Notebook" can be accessed by right-clicking on the table row corresponding to the selected molecule. This software function allows users to upload *pdf* documents, images (in *png*, *jpg* and *jpeg* formats) and text files in *txt* format. The attached files are associated with the molecule entry and easily identifiable by the paperclip icon in the attachment column found in the project table. These files are automatically stored in the MolBook UNIPI project and they can be accessed by simply reopening the "Chemical Notebook" of the corresponding molecule.

Chemical properties calculation

In order to provide useful functions for the management and analysis of chemical databases, MolBook UNIPI contains several functions to derive and predict molecular properties. For each molecule within a project, the following properties can be directly calculated and stored: molecular weight, chemical formula, number of heavy atoms, rotatable bonds, HB-Donors, HB-Acceptors, topological polar surface area (TPSA) and LogP.[4] These properties facilitate the analysis of the data set, providing an overview of the chemical features of the molecules. Chemical properties are used to calculate the Lipinsky's Rule of Five (RO5)[5] and its variant, the Rule of Three (RO3),[6] which provide a method of assessing drug-likeness and lead-likeness, respectively. These indices facilitate the user in filtering compounds according to properties that may affect pharmacokinetics and lead optimization. For a clear evaluation, the software indicates the parameters that are not met if a compound does not pass the RO5 and/or RO3 filters. Finally, the software includes the functionality to identify pan-assay interference compounds (PAINS).[2] The identification of PAINS is based on a substructure search that verifies the presence of patterns that could generate interferences during biological assays. MolBook UNIPI offers the possibility to apply the PAINS filter using three different libraries of chemical patterns. The user has the faculty to select the desired combination of libraries to be used in the substructure search. The software performs the analysis and returns the results for each selected library, indicating whether or not each compound passed the filter and reporting the number of alerts detected. In addition, the software includes a tool that allows the visualization of PAINS-related substructures. This tool is presented as a grid view window where the structure of selected molecules in the project table are displayed. The user can select one of the three available PAINS filters to highlight the matched interference substructure fragments, if present.

Toxicity predictions

VenomPred platform for *in silico* toxicity predictions, recently developed by our team,[7] was integrated into the software. The platform employs machine learning (ML) models trained with

experimentally evaluated toxicological data to predict the potential toxicity of chemical compounds in relation to four endpoints: mutagenicity, carcinogenicity, hepatotoxicity and estrogenicity. The training and test set compounds respectively used to train and evaluate the models related to all endpoints were retrieved from VEGA, a freely available toxicity assessment software. The compounds were converted into molecular fingerprints to provide binary vectors suitable for ML model fitting. For each endpoint, the compounds were represented by 5 different chemical fingerprints (FPs). The FPs were then combined with four ML algorithms, yielding 20 different models per endpoint, whose hyperparameters were properly optimized. The models were subjected to internal cross-validation and external test set validation. For each endpoint, our models achieved better or comparable performance with respect to the reference models included in VEGA. In order to improve the performance of the ML models, we applied a consensus strategy that combined the predictions of multiple models. Such strategy demonstrated to achieve a higher predictive performance; therefore, the best model combination for each endpoint was included in VenomPred platform. VenomPred returns a probability value in the range between 0 and 100 indicating the potential toxicity of small molecules in relation to a specific endpoint. A compound is classified as toxic if the probability is equal to or greater than 50, while a probability below 50 indicates a non-toxic profile. Precisely, a value closer to 100 corresponds to a highly confident prediction of potential toxicity. Similarly, a probability close to 0 represents a non-toxic prediction with high confidence. In this context, it is relevant to mention that the speed of the toxicological predictions performed by VenomPred through MolBook UNIPI depends on the hardware specifications of the computer running the software.

Query search

MolBook UNIPI projects can be easily queried to retrieve entries of molecules that have certain properties calculated by the software or added by the user, as well as to obtain compounds that have a structural similarity with respect to a molecule defined as a query. The "Property Query" widget allows user to define several queries that are applied to filter compounds and to identify only those

whose properties satisfy all the criteria specified by the user. A query on a single property is defined by three parameters: the property of the molecule to be examined, the comparison criteria, and the query value. The results are displayed in a new project that preserves the properties associated with each molecule retrieved through the query. A project can also be queried in terms of chemical structure matching with the "Structural Query" widget. By using this widget, it is possible to draw, using JSME sketcher, the chemical structure of a molecule that is used as a reference (query) to perform the comparison with the compounds in the project. Three search approaches are available for the "Structural Query": similarity, substructure and superstructure. The similarity principle is based on converting the compounds to be examined into a chemical fingerprint. MolBook UNIPI employs the RDKit library function to calculate Morgan fingerprints,[8] setting the vector length to 2048 bits and the atom radius to 2. Fingerprint similarity is calculated with the Tanimoto index,[9] which returns a value between 0 and 1, where 1 indicates that two vectors correspond to the same compound. A vertical slider is present in the "Structural Query" widget for setting the similarity threshold, which is shown in percentage values. The substructure approach relies on searching for the presence of the query molecule in the chemical structure of the compounds in the project. On the other hand, the superstructure search method identifies molecules whose structure represents a part of the structure specified as a query. Both methods are performed through the built-in RDKit molecule objects methods. Analogously to the "Property Query," the results of the "Structural Query" are displayed as new projects browsable in the corresponding tabs of the main software window.

REFERENCES

(1) Sorokina, M.; Merseburger, P.; Rajan, K.; Yirik, M. A.; Steinbeck, C. COCONUT Online: Collection of Open Natural Products Database. *J. Cheminform.* **2021**, *13* (1), 2. https://doi.org/10.1186/s13321-020-00478-9.

(2) Baell, J. B.; Holloway, G. A. New Substructure Filters for Removal of Pan Assay Interference Compounds (PAINS) from Screening Libraries and for Their Exclusion in Bioassays. *J. Med. Chem.* **2010**, *53* (7), 2719–2740. https://doi.org/10.1021/jm901137j.

(3) Landrum, G. RDKit: Open-Source Cheminformatics. Available Online: Https://Www.Rdkit.Org (Accessed on 1 February 2023).

(4) Wildman, S. A.; Crippen, G. M. Prediction of Physicochemical Parameters by Atomic Contributions. *J. Chem. Inf. Comput. Sci.* **1999**, *39* (5), 868–873. https://doi.org/10.1021/ci990307l.

(5) Lipinski, C. A.; Lombardo, F.; Dominy, B. W.; Feeney, P. J. Experimental and Computational Approaches to Estimate Solubility and Permeability in Drug Discovery and Development Settings 1PII of Original Article. *Adv. Drug Deliv. Rev.* **2001**, *46* (1–3), 3–26. https://doi.org/10.1016/S0169-409X(00)00129-0.

(6) Jorgensen, W. L. Efficient Drug Lead Discovery and Optimization. *Acc. Chem. Res.* **2009**, *42* (6), 724–733. https://doi.org/10.1021/ar800236t.

(7) Galati, S.; Di Stefano, M.; Martinelli, E.; Macchia, M.; Martinelli, A.; Poli, G.; Tuccinardi, T. VenomPred: A Machine Learning Based Platform for Molecular Toxicity Predictions. *Int. J. Mol. Sci.* **2022**, *23* (4), 2105. https://doi.org/10.3390/ijms23042105.

(8) Rogers, D.; Hahn, M. Extended-Connectivity Fingerprints. *J. Chem. Inf. Model.* **2010**, *50* (5), 742–754. https://doi.org/10.1021/ci100050t.

(9) Fligner, M. A.; Verducci, J. S.; Blower, P. E. A Modification of the Jaccard–Tanimoto Similarity Index for Diverse Selection of Chemical Compounds Using Binary Strings. *Technometrics* **2002**, *44* (2), 110–119. https://doi.org/10.1198/004017002317375064.
